# The application of graphene in lithium ion battery electrode materials

**DOI:** 10.1186/2193-1801-3-585

**Published:** 2014-10-08

**Authors:** Jiping Zhu, Rui Duan, Sheng Zhang, Nan Jiang, Yangyang Zhang, Jie Zhu

**Affiliations:** School of Materials Science and Engineering, Hefei University of Technology, Hefei, Anhui 230009 P R China

**Keywords:** Graphene, Lithium ion battery, Electrode materials, Electrochemical characterizations

## Abstract

Graphene is composed of a single atomic layer of carbon which has excellent mechanical, electrical and optical properties. It has the potential to be widely used in the fields of physics, chemistry, information, energy and device manufacturing. In this paper, we briefly review the concept, structure, properties, preparation methods of graphene and its application in lithium ion batteries. A continuous 3D conductive network formed by graphene can effectively improve the electron and ion transportation of the electrode materials, so the addition of graphene can greatly enhance lithium ion battery’s properties and provide better chemical stability, higher electrical conductivity and higher capacity. In this review, some recent advances in the graphene-containing materials used in lithium ion batteries are summarized and future prospects are highlighted.

## 1 Introduction

Nowadays, ever-increasing demands on energy have driven many countries to invest heavily in finding new sources of energy or investigating new ways/devices to store energy (Zhu et al. 2014). A kind of energy storage device is lithium ion batteries, which have many unique advantages in comparison to conventional batteries. These merits include high open-circuit voltage, high energy density, long useful life, no memory effect, no pollution and low self-discharge rate. The advantageous properties of lithium ion batteries make them quickly become the new generation of secondary batteries in recent years and they are now widely used in mobile phones, laptops and other portable electronic devices (Tarascon & Armand 2001). In lithium ion batteries, lithium ions move from the negative electrode to the positive electrode during discharge, and this is reversed during the charging process. Cathode materials commonly used are lithium intercalation compounds, such as LiCoO_2_, LiMn_2_O_4_ and LiFePO_4_; anode materials commonly used are graphite, tin-based oxides and transition metal oxides. However, these materials have some drawbacks that limit their use. For example, carbon materials have good cycle performance but low initial charge and discharge efficiency; tin-based oxides have good cycle but high irreversible capacity loss in the first cycle (Simon & Gogotsi 2008). One of the potential solutions to these problems is to develop new electrode materials for lithium ion batteries. Graphene, a miracle material, is chemically stable and has high electrical conductivity. So it has naturally been considered as a suitable electrode alternative in the battery applications (Atabaki & Kovacevic 2013).

**Figure 1:**
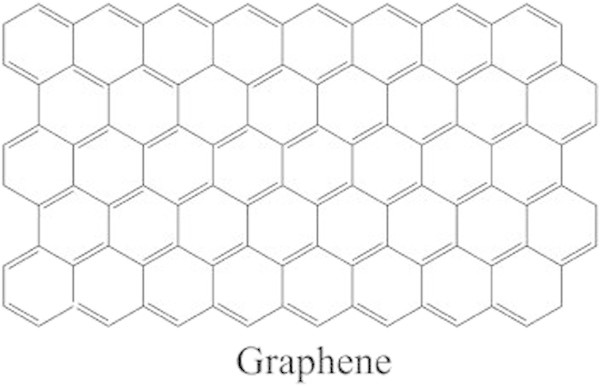
**The structural model of graphene.**

Graphene is a monolayer of graphite, consisting of sp^2^ hybridized carbon atoms arranged in a honeycomb crystal lattice (Geim & Novoselov 2007), as shown in Figure 1. It is a two-dimensional material, meaning that every atom of graphene can be considered as a surface atom. Graphene forms the basic structure of other carbon materials like graphite, carbon nanotubes and fullerenes. In 2004, Andre Geim and Kostya Novoselov obtained graphene via a simple method (Novoselov et al. 2004), which subsequently attracted attention around the world, owing to graphene’s novel structure and properties. For example, this two-dimensional carbon material has a specific surface area of 2600 m^2^/G (Stoller et al. 2008) with its honeycomb structure potentially resulting in higher lithium storage capacity. Furthermore, its high electron mobility (15000 cm^2^/(V · s)), outstanding thermal conductivity (3000 W/(m · K)) (Bolotin et al. 2008), good chemical stability and excellent mechanical properties make it an ideal target for forming composite materials used as the base electrode. Improved electrodes also allow for the storage of more lithium ions and increase the battery’s capacity. As a result, the life of batteries containing graphene can last significantly longer than conventional batteries (Bolotin et al. 2008). In the conventional lithium ion batteries, as lithium ions are inserted and removed from the electrode materials, the materials will swell and shrink, leading to a quicker breakdown. This can be avoided through the addition of graphene, whose efficient conductivity can lead to less resistive heating within the electrode, so batteries can operate at lower temperatures, which ultimately improves the battery’s safety (Atabaki & Kovacevic 2013). Graphene has many additional properties such as the quantum hall effect, bipolar field-effect, ferromagnetism, superconductivity and high electron mobility (Katsnelson et al. 2006). These properties make graphene suited for use in many fields. Moreover, recent scientific advances have allowed for the development of various low-cost and simple methods of preparing graphene. This is particularly important for large-scale production and applications. Below, a review of the applications of graphene and graphene-based composites as electrode materials in lithium ion batteries are analyzed, as well as likely paths for future development.

## 2 Preparation methods of graphene

Graphene are currently produced by several different methods: micromechanical exfoliation of highly oriented pyrolytic graphite with or without previous processing of the surface (Tang & Hu 2012; Lu et al. 1999; Fredriksson et al. 2009), epitaxial growth (Yu et al. 2011; Berger et al. 2006), chemical vapor deposition (CVD) (An et al. 2011; Wintterlin & ML 2009) and reduction of graphene oxide (GO) (Li et al. 2008; Gómez-Navarro et al. 2007; Stankovich et al. 2007; Mattevi et al. 2009; Fernandez-Merino et al. 2010). The graphene produced by micromechanical exfoliation and chemical vapor deposition shows good monolayer morphology. However these methods are complex and can only produce small amounts of grapheme, hence are not suitable for mass production and application. Chemical reduction of graphene oxide is currently the most suitable method for large-scale graphene production. So graphene used in the vast majority of lithium ion battery electrode materials is obtained by reducing GO.

Graphene oxide is produced from natural graphite through the Hummers method (Fan et al. 2008; Gómez-Navarro et al. 2007), Brodie method (Brodie & Chim 1860) or Staudenmaie method (Staudenmaier & Deut 1898). The Hummers method is most commonly used. Once GO is produced, hydrazine hydrate, NaBH_4_ (Shin et al. 2009) or other reducing agents are used to produce graphene. This mode of preparation is simple and will enable mass production of graphene. The shortfall of this method is that introduced oxygen will affect the produced graphene’s electrochemical properties, resulting in deterioration of graphene. Despite these drawbacks, chemical reduction of GO is still the primary method used by researchers, owing to its simplicity and lower equipment burden.

## 3 Graphene in lithium ion battery cathode materials

Some of the most commonly studied cathode materials used in lithium ion batteries (LIBs) are LiCoO_2_, LiMn_2_O_4_, LiFePO_4_ and Li_3_V_2_(PO_4_)_3_. These materials have electronic conductivities of 10^-4^ S/cm (Dokko et al. 2001; Barker et al. 1996; Levasseur et al. 2002), 10^-6^ S/cm (Marzec et al. 2002; Cao & Prakash 2002), 10^-9^ S/cm (Prosini et al. 2002; Shi et al. 2003) and 2.4 × 10^-7^ S/cm (Pan et al. 2011) respectively. These electronic conductivity values are fairly low when high battery performance is required, so electron conducting additives are frequently added to such materials in order to improve their electrochemical properties.

Existing studies show that pure graphene can’t become a direct substitute for current carbon-based commercial electrode materials in lithium ion batteries due to its low coulombic efficiency, high charge–discharge platform and poor cycle stability (Atabaki & Kovacevic 2013). However, when used as a matrix in the composite electrode materials, graphene can play a very important role.

In recent years, researchers have begun to study graphene modified for use as a cathode material and have found that it can significantly improve cathode electrochemical performance (Geim & Novoselov 2007). For example, the two-dimensional large surface area and superior electron transfer capability of graphene can effectively improve the transmission and diffusion abilities of electron and ion in cathode materials.

### 3.1 Lithium metal oxide-graphene composites as cathode materials for LIBs

LiMn_2_O_4_ is used as cathode electrode material, owing to its low cost, environmental friendliness and high abundance (Manev et al. 1995). However, its low electrical conductivity results in a low-rate capacity. Published papers have demonstrated that graphene sheets are effective agents for improving their conductivity and rate capacity. LiMn_2_O_4_-graphene composites with high rate capacity were synthesized by a microwave assisted hydrothermal method (Bak et al. 2011). The composites exhibited reversible capacities of 117 mAh/g and 101 mAh/g at 50C and 100C. In another study, LiMn_2_O_4_-graphene composites were synthesized by self-assembly approach combined with a solid-state lithiation method (Zhao et al. 2011). The enhancement in electrochemical properties is attributed to the superior Li^+^ diffusion kinetics and improved stability across a wide voltage range in crystalline LiMn_2_O_4_-graphene composites. Furthermore, their capacities approached the theoretical value and the cycling stability was enhanced.

LiNi_1/3_Mn_1/3_Co_1/3_O_2_ is a promising candidate for cathode electrode materials. It shows high energy density, good stability, enhanced safety and can be produced at low cost (Zhu et al. 2012). However, cation disorder occurs during calcination and results in deterioration of its kinetic properties. To improve its electrochemical performance, LiNi_1/3_Mn_1/3_Co_1/3_O_2_-graphene composites are prepared as cathode materials for LIBs. Jiang and coworkers reported that LiNi_1/3_Mn_1/3_Co_1/3_O_2_-graphene composites prepared by mechanical mixing could deliver a capacity of 115 mAh/g at 6C (Jiang et al. 2012). LiNi_1/3_Mn_1/3_Co_1/3_O_2_-graphene composites prepared by micro-emulsion and ball-milling route could deliver a reversible capacity of 150 mAh/g at 5C, much higher than that of bare LiNi_1/3_Mn_1/3_Co_1/3_O_2_ (Rao et al. 2011). The improved performance is attributed to grain connectivity and high electronic conductivity.

### 3.2 LiMPO_4_-graphene composites as cathode materials for LIBs

**Figure 2:**
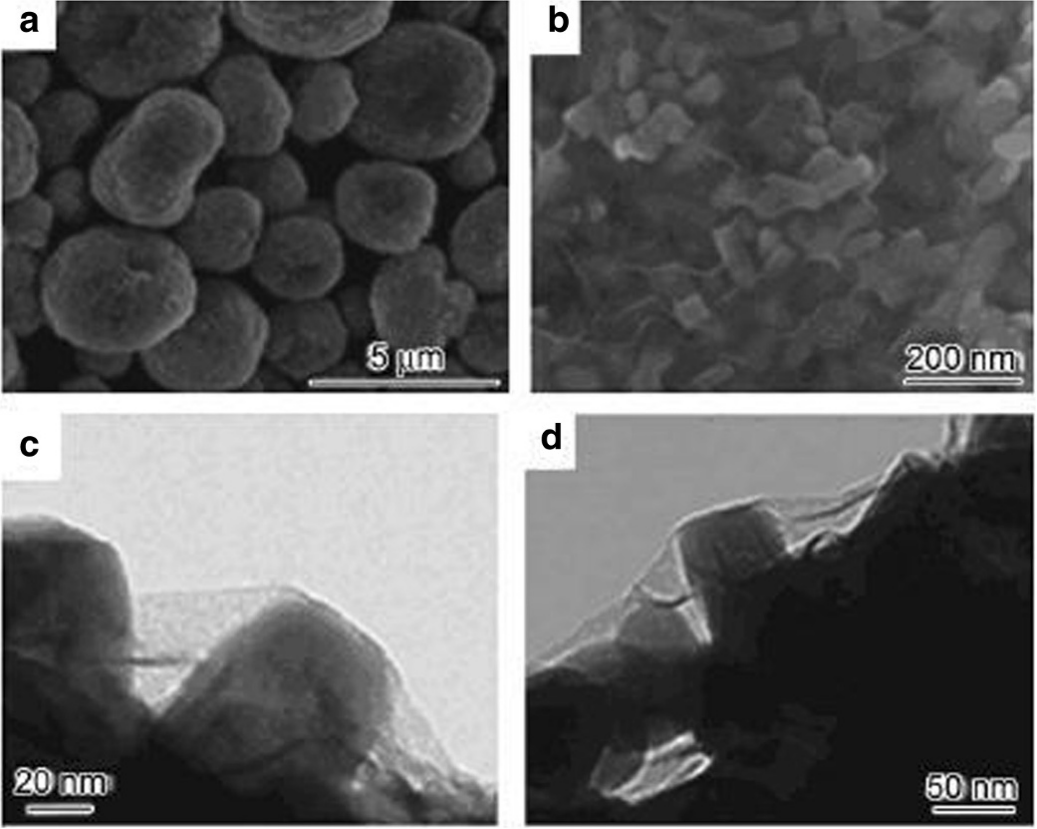
**SEM and TEM images of the composite. (a, b)** SEM images showing an overview of the LFP /G particles. **(c)** TEM image illustrating a local area of one LFP nanoparticle in an LFP/G secondary particle. **(d)**TEM image showing a local area of one LFP nanoparticle in an LFP/(G + C) secondary particle

When used as an electrode material, LiFePO_4_ has the advantages of high specific capacity 170mAh/g, low cost and low toxicity (Kobayashi et al. 2009). However, its low electrical conductivity (10^- 9^S/cm^2^) and poor lithium ion diffusion (10^- 14^ -10^- 16^ cm^2^/S) lead to capacity fade quickly under high rate charge and discharge (Amin & Maier 2008). The decision to add graphene to improve the electric properties of the phosphate was based on following premises. Firstly, graphene's high conductivity could enhance the conductance of electrode materials. Secondly, the mechanical properties of graphene could help maintain the microstructure of the phosphate and improve cyclic stability. Indeed, for the LiFePO_4_/graphene composite material, the flexible mesh structure of graphene improved its electrical conductivity and ratio performance. The LiFePO_4_/graphene nanocomposite was prepared by various synthesis routes, with hydrothermal, solvothermal and solid state routes (Amin & Maier 2008). In addition, the researcher also generated LiFePO_4_ (LFP) nanoparticles, mixed them with graphite oxide in solution. The mixed solution was then underwent a spray-dried and sintered process to obtain the LiFePO_4_/graphene composite (LFP/G) shown in Figure 2. The result showed that graphene well coated the surface of the LiFePO_4_, with a thickness of approximately 2 nm (about 3–5 layers of graphene) and formed a continuous lamellar structure; LiFePO_4_ nanoparticles had uniform size at 2–5 nm. The obtained material displays a more regular morphology and structure, which can potentially lead to a large enhancement of conductivity. In addition, this material can be further carbon coated to obtain carbon coated LiFePO_4_/graphene (LFP/(G + C)), which presents superior rate and cycle performances.

In comparison to LiFePO_4_, Li_3_V_2_(PO_4_)_3_ is an attractive cathode material for LIBs, because its average extraction/reinsertion voltage is about 4.0 V, and its theoretical capacity is 197 mAh/g (Huang et al. 2009; Yu et al. 2012). Li_3_V_2_(PO_4_)_3_ forms a monoclinic structure and has a high operating voltage and shows a good performance at high discharge currents. However, its intrinsic low electronic conductivity (240 nS/cm at 25°C) limits its rate capacity, so graphene is added to improve its electrochemical performance. Li_3_V_2_(PO_4_)_3_/graphene cathode material has been prepared by sol–gel, solid state and spray-drying synthesis methods (Huang et al. 2009; Yu et al. 2012). The product which was prepared by a sol–gel route shows excellent rate capacity and cycling stability (Yu et al. 2012).

## 4 Graphene in lithium ion battery anode materials

Graphene has opened new possibilities in the field of lithium ion battery materials due to its light weight, high electrical conductivity, superior mechanical flexibility, and chemical stability (Su et al. 2012). These properties prove advantageous when graphene is used in the anode. The addition of graphene to anode materials has lead to superior electrical conductivity, high surface area (2620 m^2^g^-1^), high surface-to-volume ratio, ultra-thin thickness which can shorten the diffusion distance of ions, structural flexibility that paves the way for constructing flexible electrodes, thermal and chemical stability which guarantee its durability in harsh environments.

At present, non-carbon-based lithium-ion battery anode materials are mainly tin-based electrode materials, as well as silicon-based and transition metal-based materials (Zhu et al. 2011; Liu et al. 2012; Wang et al. 2010a; Lian et al. 2010a; Tao et al. 2012; Wang et al. 2010b; Kim et al. 2012; Tung et al. 2009; Cai et al. 2012a; Wang et al. 2011a). Even though the aforementioned materials have high theoretical capacity, drawbacks to their use as anode materials are volume expansion during lithium/delithiation and a large internal stress. After repeated charging and discharging, the material is prone to rupture, resulting in poor cycling performance. To overcome these disadvantages, graphene is adopted. Table 1 summarizes LIB anode materials (non-carbon) doped with graphene. Some widely and commonly used materials are discussed in this paper.

**Table 1:** **Summary of LIB anode materials (non-carbon) containing graphene**

Anode materials	Structure	Synthesis method	Capacity and cycle performance	Reference
SnO_2_/graphene	orthorhombic	Hydrothermal	First discharge capacity 1588 mAhg^-1^, after 40 cycles remain 730 mAh/g	(Zhu et al. 2011)
Si/graphene	Cubic diamond type	Hydrazine reduction	First discharge capacity 2753 mAhg^-1^, after 50 cycles remain 590 mAh/g	(Liu et al. 2012; Wang et al. 2010a)
Co_3_O_4_/graphene	spinel	Solvothermal	First discharge capacity 1826 mAhg^-1^, after 40 cycles maintain 1310 mAh/g	(Lian et al. 2010a)
Mn_3_O_4_/graphene	spinel	Hydrothermal	First discharge capacity 900 mAhg^-1^, after 100 cycles maintain 390 mAh/g	(Tao et al. 2012)
CuO/graphene	sphalerite	N-methyl-2-p yrrolidone solvent	First discharge capacity 640 mAhg^-1^, after 50 cycles maintain 583.5 mAh/g.	(Wang et al. 2010b)
Fe_3_O_4_/graphene	Trans spinel	Reduction	First discharge capacity 1426 mAhg^-1^, after 100 cycles maintain 580 mAh/g	(Kim et al. 2012)
TiO_2_/graphene	Rutile type	Gas/liquid interface reaction	First discharge capacity 499 mAhg^-1^, after 10 cycles maintain 150 mAh/g	(Tung et al. 2009)
CeO_2_/graphene	Face-centered cubic	Hydrothermal	First discharge capacity 1469 mAhg^-1^, after 100 cycles maintain 605 mAh/g	(Cai et al. 2012a)
SnS_2_/graphene	Hexagonal crystal structure	Solution phase method	First discharge capacity 1664 mAhg^-1^, after 500 cycles maintain 600 mAh/g	(Wang et al. 2011a)
Fe_3_O_4_-SnO_2_-gra-phene	——————	Gas–liquid interfacial reaction	First discharge capacity 1740 mAhg^-1^, after 115 cycles maintain 1198 mAh/g	(Chang et al. 2012)
Li_4_Ti_5_O_12_/graphe-ne	Spinel	Sol–gel method	First discharge capacity 430 mAhg^-1^, after 35cycles maintain 150 mAh/g	(Lian et al. 2011; Choucair et al. 2009a)

### 4.1 Graphene modified tin-based oxide

Sn and their oxides such as SnO_2_ are exclusively studied as lithium ion battery anode materials. However, their use is limited by a defect, in that chemical reduction will often bring in electronic barriers to Li^+^ repulsion. Lithiation and delithiation reactions (Sn + 4.4Li^+^+ 4.4e^-^↔Li_4.4_Sn) can cause large volume changes. This leads to the pulverization of the particles and the electrical disconnection of the electrode. In order to circumvent this, new anode materials with graphene have been examined in many recent studies. For example, the performance of anode electrode was improved when tin nanoparticles embedded in grapheme was used (Liang et al. 2011).

The graphene matrix not only can accommodate the volume change of Sn during charge–discharge, but also facilitate electron transport because of its high electronic conductivity. To prepare this type of anode, it is essential to use hydrothermal synthesis and subsequent annealing. It is reported that the reversible specific capacity of the nanocomposite is 662 mAhg^-1^ at a specific current of 100 mAg^-1^ after 100 cycles, and 417 mAhg^-1^ at the high current of 1000 mAg^-1^(Liang et al. 2011).

**Figure 3:**
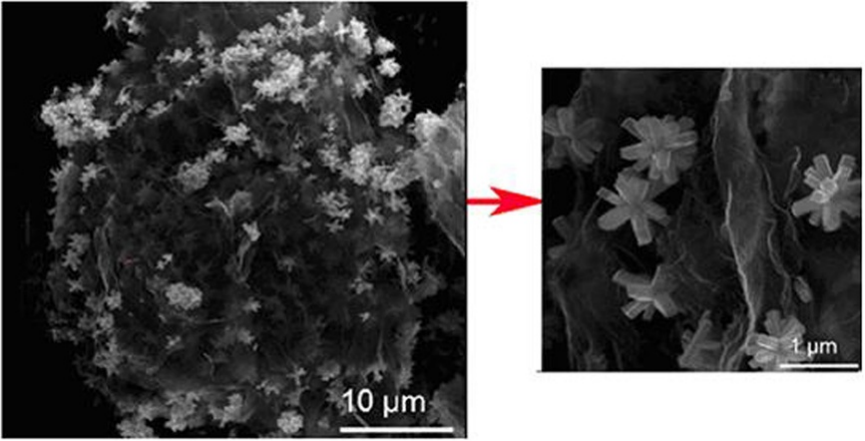
**SEM image of flower-like SnO**_**2**_ **-graphene in different magnifications.**

The SnO_2_/graphene composite can also be synthesized by a simple hydrothermal method for high-capacity lithium storage. Flower-like SnO_2_ nanorod clusters with a size of 800 nm are the product of the synthesized process (Figure 3). The flower-like SnO_2_/graphene composite shows a first discharge and charge capacity of 1588 mAhg^-1^and 1240 mAhg^-1^ at the current density of 50 mAg^-1^, respectively (Evanoff et al. 2011). After 40 cycles at different current densities of 50, 100, and 500 mAg^-1^, the reversible discharge capacity was still maintained at 730 mAhg^-1^.

### 4.2 Graphene-modified silicon-based materials

As an anode material, silicon and lithium ions can form Li _4.4_ Si. The theoretical charge capacity of this compound is up to 4200 mAh/g, it also has a low discharge voltage. However, a limitation to its use is its charge volume effect. During the discharge process, silicon and lithium form Li_3.75_Si. As a result, Si volume increased up to 270%. This leads to poor circulation stability (Wolfenstine 1999). The addition of Silicon nanomaterials and carbon-coating can buffer this volume expansion to some extent. When graphene is introduced, it can not only prevent silicon nanoparticles gathering but also improve the electron and lithium ions transport capability.

Yushin etc. (Evanoff et al. 2011) used a vapor deposition method to form a continuous Si film on the graphene sheet surface. Following that, a high temperature treatment in propylene allowed the silicon surface to be coated with carbon. The obtained composite material showed enhanced conductivity as well as oxidation resistance. This composite has 3D porous structure, which buffer Si volume change during charge and discharge, owing to the presence of a stable solid electrolyte interface film. Furthermore, this composite has more than 1000 mAh/g specific delithiated capacity and good cycle stability under 1400 mA/g current density.

### 4.3 Graphene modified transition metal-based materials

Transition metal oxides which have high lithium storage capacity are known as potential alternative anode materials for high capacity lithium ion batteries. Owing to the presence of volume changes during charge and discharge and their low conductivity, graphene can also be used to improve their electrochemical properties.

Co_3_O_4_ has a high theoretical capacity of 890mAh/g. However, the processes of charging and discharging cause large volume expansion. The addition of graphene can effectively improve Co_3_O_4_ electrochemical properties (Kim et al. 2011; Li et al. 2011; Yan et al. 2010; Wu et al. 2010; Yang et al. 2010). For example, at 200 mA/g of current density, Co(OH)_2_’s first cycle off-lithium specific capacity is 660 mAh/g. Through synchronizing hydrothermal reduction with graphene, the material’s delithiated specific capacity increases up to 1120mAh/g. After 30 cycles, the reversible capacity of the composite material remains 82% of the initial capacity.

Mn_3_O_4_ has a 936 mAh/g theoretical capacity. However, due to its poor electrical conductivity (about 10^- 7^-10^- 8^ S/cm), the actual capacity with Co doped can only reach a maximum of 400 mAh/g. When the composite with graphene formed through a two-step liquid method, followed by hydrothermal synthesis, the delithiated ratio capacity of this compound is about 900 mAh/g at low current density (40 mA/g), closing to the theoretical capacity. When current density reaches 1600 mA/g, the specific capacity of this compound maintains 390 mAh/g (Wang et al. 2010c).

CuO has low band gap energy and high catalytic activity. However, as an anode material, it has a low conductive performance and large volume expansion effect. These shortcomings can be improved by forming CuO/graphene composites (Mai et al. 2011). First CuO and graphite oxide are used to produce CuO/graphene composite by hydrothermal synthesis, followed by a reduction process. After 50 cycles, the composite material’s inverse capacity reached 583.5 mAh/g and the capacity retention ratio was 75.5%.

Fe_3_O_4_/graphene composites have excellent high-rate performance, except when prepared by gas–liquid interface method (Lian et al. 2010b). At 35 mA/g current density, the specific reversible capacity holds at 1026 mAh/g (30 cycles). At 700 mA/g current density, the capacity is still 580 mAh/g after 100 cycles. Fe_2_O_3_ is used as anode material owing to its high theoretical capacity (1005 mAh/g) and low price (Choucair et al. 2009a). (Wang et al. 2011b) prepared Fe_2_O_3_/graphene composites by a hydrothermal method. The addition of graphene prevented Fe_2_O_3_ from aggregation and also buffered material’s volume expansion. At l160 mA/g current density, the reversible capacity of the composite remains 660 mAh/ g after 100 cycles.

TiO_2_ with graphene is another nanocomposite that can be synthesized by a facile gas/liquid interface reaction (Cai et al. 2012a). The electrochemical performance tests show that the composite’s specific charge capacity is 499 mAh g^-1^ at a current density of 100 mAg^-1^. This specific charge capacity drops to 150 mAhg^-1^at a high current density of 3000 mAg^-1^. The advantage of this nanocomposite is that the oxygen-containing groups on the graphene sheets can be reduced after the heat treatment and residual functional groups including the OH and COOH on the surface of graphene sheets can strongly interact with the metal ions during the synthesis process .The random hybridization of TiO_2_ nanoparticles and ultrathin graphene sheets form a three-dimensional porous structure of the TiO_2_-graphene nanocomposite. The small nanoparticles provide a short mean-free-path for electron and lithium ion to travel during the lithium ion insertion/extraction process, resulting in excellent rate capability.

## 5 Conclusion

Compared to traditional carbon electrode materials, graphene, due to its low initial coulombic efficiency, high charge and discharge platform and other shortcomings, cannot directly replace traditional graphite as an anode material. However, graphene does demonstrate high electrical conductivity, good mechanical strength, excellent flexibility, great chemical stability and high specific surface area. This is especially noticeable when graphene is chemically converted with a greater proportion of functional groups, proving that it is suited for use as a base composite electrode material. When used as electrode material, graphene can effectively reduce the size of the active material, prevent agglomeration of nanoparticles, improve electrons and ions transmission capacity, as well as enhancing the electrode’s mechanical stability. As a result, graphene-containing electrode materials have high capacity and good rate performance. Lithium metal oxide-graphene, LiMPO_4_-graphene, Tin-based, Si-based and transition metal based electrode materials with graphene have been extensively studied in this paper. The composite materials’ advantages can be summarized as following. Firstly, graphene’s flexibility makes it an ideal material to buffer metal electrode’s volume expansion and contraction during the charge–discharge process. This improves the electrode material’s cycle life performance. Further, the excellent electrical properties of graphene can enhance the conductivity of metal electrode material. Moreover, the addition of graphene can control the growth of metal oxide particles. Smaller particles means the diffusion distance of lithium ions and electrons is reduced, this improves the material’s rate performance. Finally, the lithium storage capacity for most metal oxide composite materials with graphene has improved greatly.

In addition, various approaches are taken to prepare graphene. These vary from simple mechanical mixing to the well controlled in situ reactions and interfacial reactions, which result in better graphene morphology and structure. Developing easily replicable methods for producing graphene is the key to future application of graphene in lithium ion batteries.
